# Recurrent triple-negative breast cancer (TNBC) tissues contain a higher amount of phosphatidylcholine (32:1) than non-recurrent TNBC tissues

**DOI:** 10.1371/journal.pone.0183724

**Published:** 2017-08-23

**Authors:** Yuko Hosokawa, Noritaka Masaki, Shiro Takei, Makoto Horikawa, Shoko Matsushita, Eiji Sugiyama, Hiroyuki Ogura, Norihiko Shiiya, Mitsutoshi Setou

**Affiliations:** 1 1st Department of Surgery, Hamamatsu University School of Medicine, Handayama, Higashi-ku, Hamamatsu, Shizuoka, Japan; 2 International Mass Imaging Center and Department of Cellular and Molecular Anatomy, Hamamatsu University School of Medicine, Handayama, Higashi-ku, Hamamatsu, Shizuoka, Japan; 3 Preeminent Medical Photonics Education & Research Center, Handayama, Higashi-ku, Hamamatsu, Shizuoka, Japan; 4 Department of Anatomy, The University of Hong Kong, 6/F, William MW Mong Block Sassoon Road, Pokfulam, Hong Kong SAR, China; 5 Riken Center for Molecular Imaging Science, Minatojima-minamimachi, Chuo-ku, Kobe, Hyogo, Japan; Florida International University, UNITED STATES

## Abstract

Triple-negative breast cancer (TNBC) is one of the breast cancer subtype that displays a high risk of early recurrence and short overall survival. Improvement of the prognosis of patients with TNBC requires identifying a predictive factor of recurrence, which would make it possible to provide beneficial personalized treatment. However, no clinically reliable predictive factor is currently known. In this study, we investigated the predictive factor of recurrence in TNBC using matrix-assisted laser desorption/ionization-imaging mass spectrometry for lipid profiling of breast cancer specimens obtained from three and six patients with recurrent and non-recurrent TNBC, respectively. The signal for phosphatidylcholine (PC) (32:1) at *m/z* 732.5 was significantly higher in the recurrence group compared to the non-recurrence group (P = 0.024). PC (32:1) was more abundant in the cancer epithelial area than it was in the surrounding stroma, suggesting that abnormal lipid metabolism was associated with malignant transformation. Our results indicate PC (32:1) as a candidate predictive factor of TNBC recurrence. A future prospective study investigating whether personalized therapy based on PC (32:1) intensity improves the prognosis of patients with TNBC is recommended.

## Introduction

Breast cancer is one of the leading causes of cancer-related deaths in women worldwide [1]. In particular, triple-negative breast cancer (TNBC) is an aggressive type associated with early recurrence after diagnosis and a short overall survival[2]. TNBC is defined by its lack of expression of oestrogen and progesterone receptors and overexpression of human epidermal growth factor receptor 2 (HER2)[2]. Owing to the absence of a targeted therapy for TNBC such as endocrine or anti-HER2 therapy, treatment is limited to chemotherapy after recurrence [2]. TNBC is known to be a heterogeneous group of cancer subtypes with variable prognosis [3]. Identifying the predictive factor of recurrence is required for improving the prognosis of patients with TNBC by providing an appropriate personalized therapy. Genomic and proteomic approaches have been generally utilized for investigating possible prognostic factors and therapeutic targets, such as *BRCA1*, *TP53*, Ki67, epidermal growth factor receptor, vascular endothelial growth factor, and vascular endothelial growth factor receptor, whereas few lipidomic approaches have been applied [4].

Lipid metabolism plays an important role in cancer progression [5]. Alterations in lipid metabolism have been reported in numerous types of cancer including breast cancer [5]. Phosphatidylcholine (PC) is the major lipid component of most eukaryotic membranes [6]. PC is also a substrate for generating second messengers that play an important role in cell signaling in cell proliferation, motility, invasion, and differentiation [7]. The fatty acid (FA) compositions of PC in breast cancer and human mammary epithelial cells vary and several enzymes involved in lipid metabolism are overexpressed in breast cancer cells [8]. The difference in the FA composition of PC in breast cancer cells has been associated with their metastatic potential [8]. The FA composition of PC in human breast cancer tissue differs from that of non-malignant breast tissue and ductal carcinoma *in situ* [9]. However, the relationship between the FA composition of PC in the primary lesions of breast cancer and recurrence is still unknown.

Matrix-assisted laser desorption/ionization (MALDI)-imaging mass spectrometry (IMS) is an efficient tool for profiling lipid composition and visualizing the distribution of lipids in tissue sections while preserving their positional information [10]. MALDI-IMS is a recently developed technique for simultaneously performing imaging-based morphological observations and mass spectrometry [10]. In the current study, we investigated a potential predictive factor of TNBC recurrence using MALDI-IMS to profile lipids by focusing on the FA composition of PC in the primary lesion of TNBC.

## Results

### Clinicopathological features

Table 1 shows the clinicopathological features of analysed tissue samples. All samples analyzed in this study were obtained from female patients (*n* = 9) with an average age of 63.7 years (range, 34–88 years). There were six invasive ductal carcinomas, two invasive lobular carcinomas, and one medullary carcinoma. No distant metastasis was found preoperatively in any patients. A recurrent case (No.8) received preoperative chemotherapy. The median follow-up period was 24 months (range, 2–49 months). Of the nine patients, three had distant metastasis that resulted in death owing to recurrent breast cancer.

**Table 1:** Clinicopathological features.

No	Age (years)	Histological Classification	Stage (TNM)	Recurrence	DFS (month)	OS (month)
1	43	ductal	StageIIA(T2N0M0)	-	49	49
2	56	lobular	StageIIIC (T3N3M0)	-	48	48
3	68	lobular	Stage I (T1N0M0)	-	41	41
4	48	ductal	Stage I (T1N0M0)	-	39	39
5	74	medullary	Stage I (T1N0M0)	-	24	24
6	88	ductal	StageIIA (T2N0M0)	-	21	21
7	75	ductal	StageIIB (T2N1M0)	+	11	29^a^
8	88	ductal	StageIIB (T2N1M0)	+	24	25^a^
9	34	ductal	StageIIIC (T3N3M0)	+	2	7^a^

TNM: Tumor-node-metastasis classification according to the seventh edition of cancer staging by the Union for International Cancer Control.

DFS: disease free survival; OS: overall survival

^a^Death by recurrent breast cancer

### Comparison of averaged signal intensities between non-recurrence and recurrence groups

**Fig 1:**
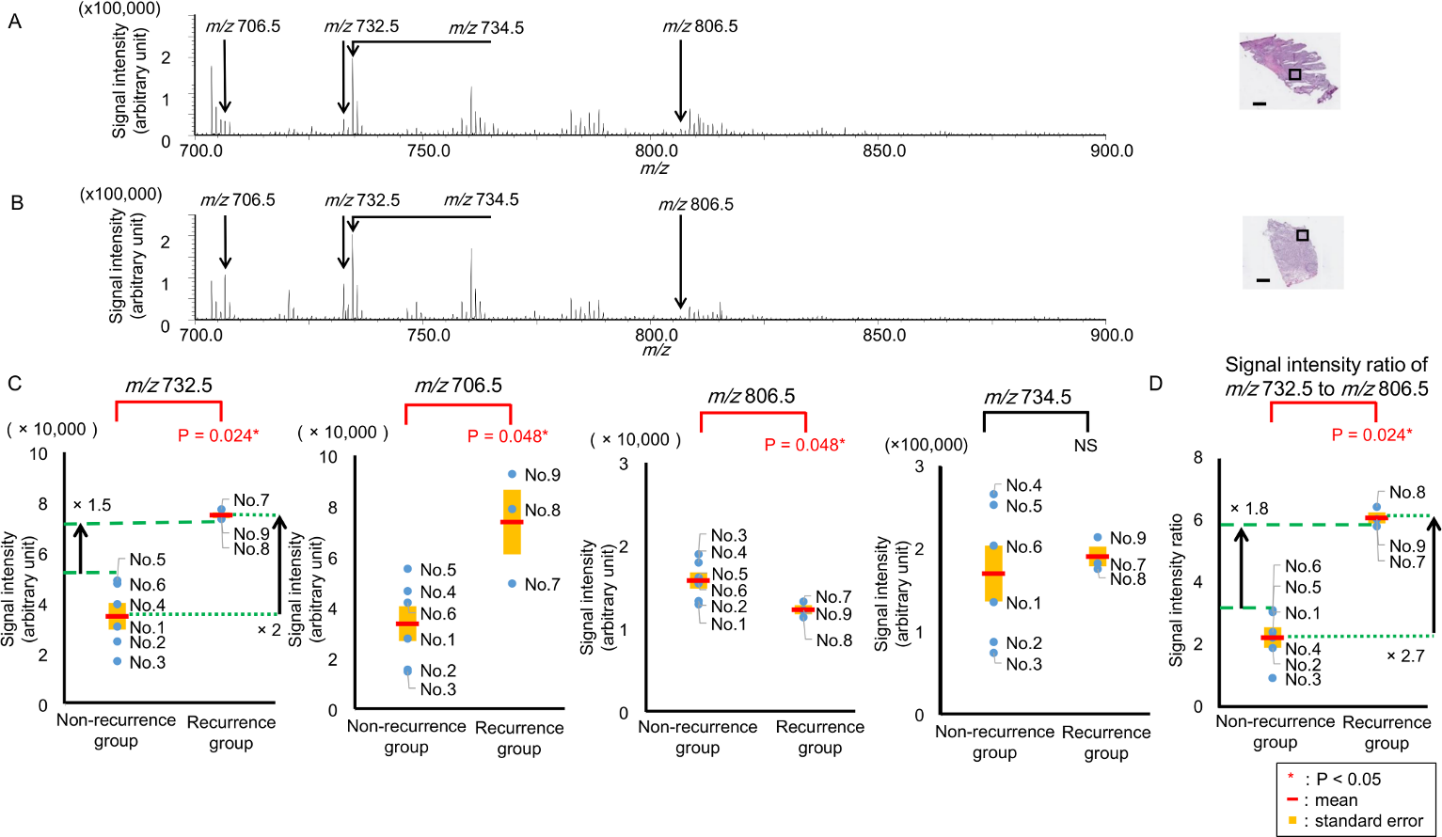
Comparison of averaged signal intensities between non-recurrence and recurrence groups. (A) Averaged mass spectrum obtained from the cancer epithelial area of the non-recurrence case (No. 1). (B) Averaged mass spectrum from the cancer epithelial area of the recurrence case (No. 9). Regions of interest set in cancer epithelial areas are presented as black squares in the hematoxylin and eosin -stained images, Scale bar = 1000 μm. (C) Averaged signal intensities of *m/z* 732.5 and 706.5 in the recurrence group (*n* = 3) were significantly higher than those in the non-recurrence group (*n* = 6, P = 0.024* and 0.048*, respectively). Averaged signal intensity of *m/z* 806.5 in the recurrence group (*n* = 3) was significantly lower than that in the non- recurrence group (*n* = 6, P = 0.048*) No significant differences occurred between the two groups related to the signal intensity at *m/z* 734.5 (P >0.5). The mean value of the averaged signal intensity at *m/z* 732.5 in the recurrence group was two times higher than that in the non-recurrence group. The lowest averaged signal intensity at *m/z* 732.5 in the recurrence group (No. 8) was 1.5 times higher than the highest in the non-recurrence group (No. 5). (D) Compared with the non-recurrence group, the ratio of the signal intensity of *m/z* 732.5 to that of *m/z* 806.5 was significantly higher in the recurrence group (P = 0.024*).* The mean value of the ratio of the signal intensity at *m/z* 732.5 to that at *m/z* 806.5 in the recurrence group was 2.7 times higher than that in the non-recurrence group. The highest ratio of the signal intensity at *m/z* 732.5 to that at *m/z* 806.5 in the recurrence group (No. 7) was 1.8 times higher than the lowest in the non-recurrence group (No. 6). P < 0.05. NS: not statistically significant.

Fig 1A and 1B show the representative mass spectra obtained from a sample of a non-recurrence case (No. 1) and a recurrence case (No. 9). Fig 1C compares the averaged signal intensities at *m/z* 732.5, 706.5, 806.5, and 734.5 between the recurrence group (*n* = 3) and the non-recurrence group (*n* = 6). Averaged signal intensity is obtained by averaging signal intensity inside a single region of interest (ROI). Compared to the non-recurrence group, the averaged signal intensities at *m/z* 732.5 and 706.5 of the recurrence group were significantly higher (Fig 1C, P = 0.024 and 0.048, respectively). In contrast, compared to the non-recurrence group, the averaged signal intensity at *m/z* 806.5 of the recurrence group was significantly lower (Fig 1C, P = 0.048). Conversely, the averaged signal intensity at *m/z* 734.5 showed no significant differences between the two groups (Fig 1C, P > 0.05). Comparison of the averaged signal intensities at *m/z* 703.5, 756.5, 758.5, 760.5, 762.5, 780.5, 782.5, 784.5, 786.5, 788.5, 808.5, 810.5, 812.5, 814.5, and 816.5 between the recurrence and non-recurrence groups are also presented in S1 Fig. There were no significant differences between the two groups (S1 Fig). Compared with the non-recurrence group, the ratio of the signal intensity of *m/z* 732.5 to that of *m/z* 806.5 was significantly higher in the recurrence group (P = 0.024, Fig 1D). The mean value of the averaged signal intensity at *m/z* 732.5 in the recurrence group was two times higher than that in the non-recurrence group (Fig 1C). The lowest averaged signal intensity at *m/z* 732.5 in the recurrence group (No. 8) was 1.5 times higher than the highest in the non-recurrence group (No. 5). The mean value of the ratio of the signal intensity at *m/z* 732.5 to that at *m/z* 806.5 in the recurrence group was 2.7 times higher than that in the non-recurrence group (Fig 1D). The highest ratio of the signal intensity at *m/z* 732.5 to that at *m/z* 806.5 in the recurrence group (No. 7) was 1.8 times higher than the lowest in the non-recurrence group (No. 6) (Fig 1D).

### Distribution analysis of the detected ions in non-recurrence and recurrence groups

**Fig 2:**
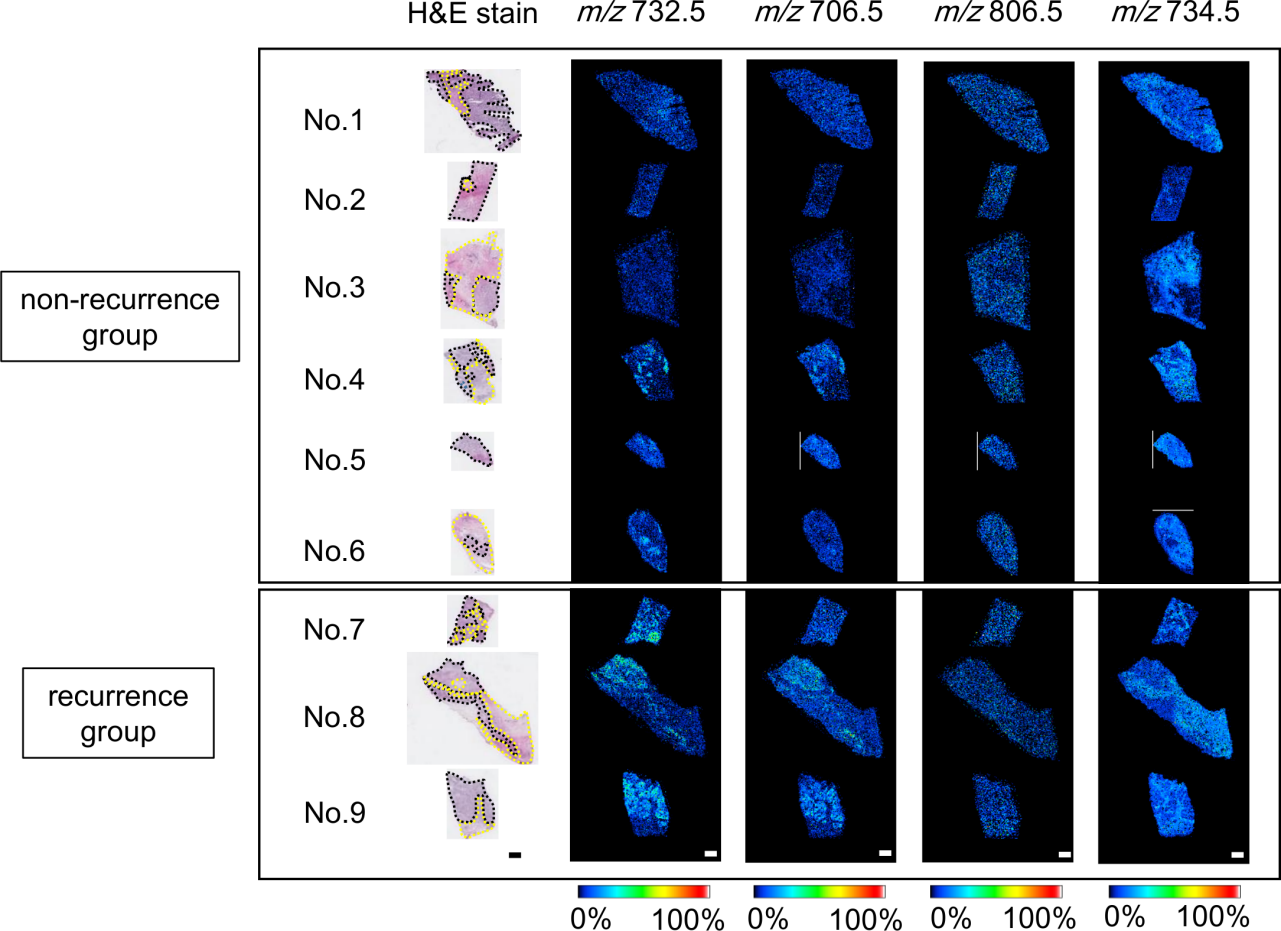
Ion distributions of non-recurrence and recurrence groups. Characteristic distributions of ions are shown in samples of non-recurrence (upper) and recurrence (lower) groups. Regions circled by black and yellow dashed lines represent the cancer epithelial area and surrounding stroma, respectively. Signal intensities at *m/z* 706.5 and *m/z* 732.5 in the cancer epithelial area of the recurrence group were higher than those of the non-recurrence group. Both groups showed higher signal intensities at *m/z* 734.5 in surrounding stroma. Scale bar = 1000 μm. MALDI-IMS: matrix-assisted laser desorption/ionization-imaging mass spectrometry; H&E: hematoxylin and eosin.

The images of the interested ions at *m/z* 732.5, 706.5, 806.5, and 734.5 in the non-recurrence cases (above) and the recurrence cases (below) are presented in Fig 2. The images of the ions at *m/z* 703.5, 756.5, 758.5, 760.5, 762.5, 780.5, 782.5, 784.5, 786.5, 788.5, 808.5, 810.5, 812.5, 814.5, and 816.5 of all samples are presented in S2 Fig. Fig 2 shows the different distribution of the ions between the cancer epithelial area and the surrounding stroma, as revealed by the hematoxylin and eosin (H&E) staining. Ions at *m/z* 706.5 and *m/z* 732.5 were similarly distributed in the cancer epithelial area in recurrence cases whereas ions at *m/z* 734.5 were abundant in the surrounding stroma of all samples (Fig 2). There were no differences between the cancer epithelial area and the surrounding stroma in ions at *m/z* 806.5 (Fig 2).

### Identification of the ions of interest using tandem MS (MS/MS) analysis

**Fig 3:**
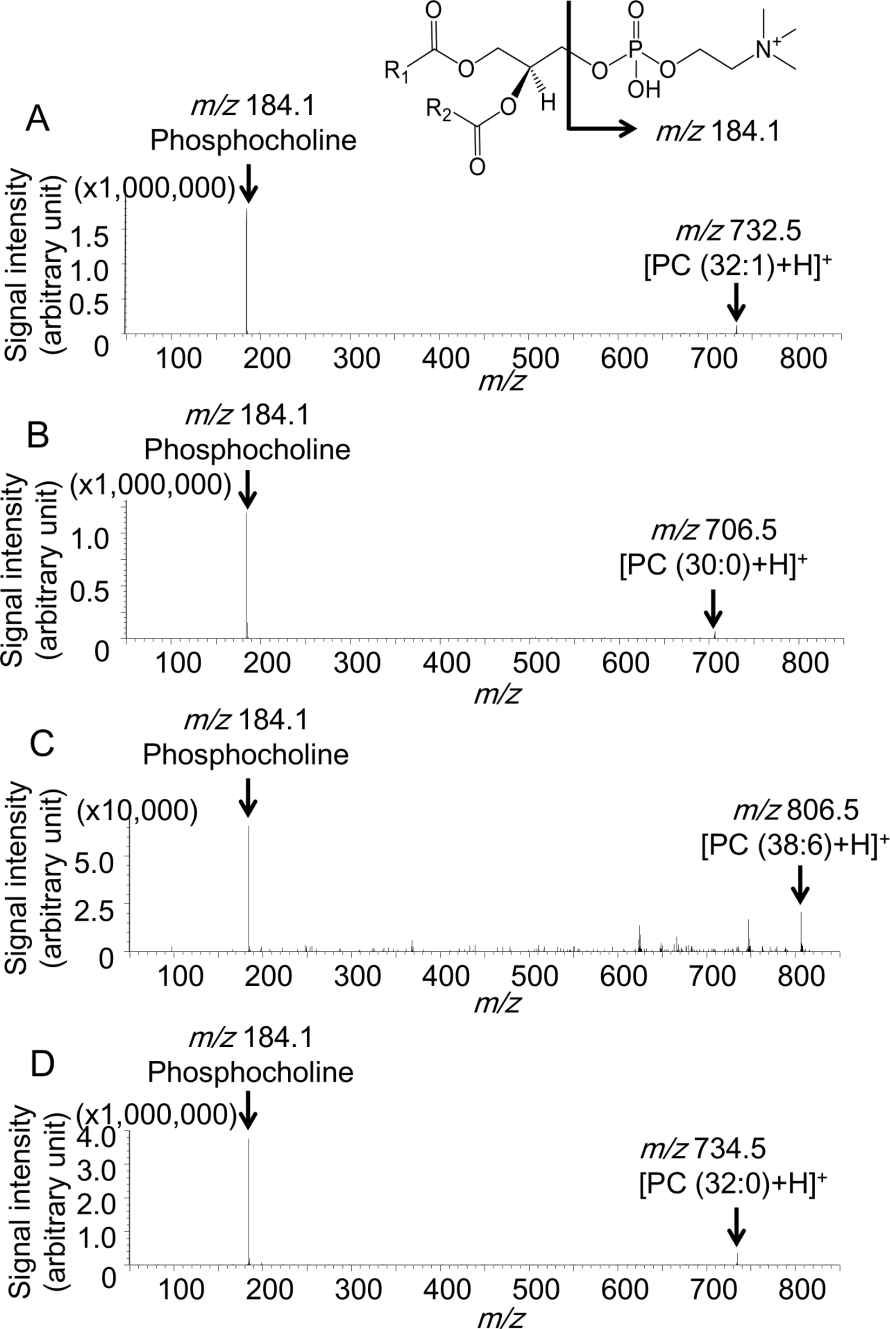
Tandem mass spectrometry for *m/z* 732.5, *m/z* 706.5, *m/z* 806.5, and *m/z* 734.5. Precursor ions for each spectrum were (A) *m/z* 732.5, (B) *m/z* 706.5, (C) *m/z* 806.5, and (D) *m/z* 734.5. The product ion spectra show a common peak corresponding to phosphocholine.

The results of the tandem MS (MS/MS) analysis for the ions at *m/z* 732.5 706.5, 806.5, and 734.5 are shown in Fig 3. An ion at *m/z* 184.1, corresponding to phosphocholine, was found as the common product ion in all the result sets [11]. This product ion and the *m/z* values of the precursor ions strongly suggest that the ions of interest were the protonated PC ions. This is consistent with the insight that the ammonium sulfate that is used in this study has the effect of reducing the generation of alkali metal ion adduct forms but enhances production of protonated ion forms for PCs. Moreover, the pattern of the protonated PCs, which is known to contain highly abundant product ions at *m/z* 184.1, is also consistent with our result [12]. According to the MS/MS results and a previous report [13], we identified the ions at *m/z* 732.5, 706.5, 806.5, and 734.5 as protonated PC (32:1), PC (30:0), PC (38:6), and PC (32:0), respectively. The other ions were identified corresponding to the previous reports and the Human Metabolome Database reference (S1 Table).

## Discussion

In this study, we examined whether any specific lipid species had a differential abundance between clinical specimens from the recurrence and the non-recurrence groups of patients with TNBC using MALDI-IMS to identify the predictive factor of recurrence in TNBC. The amounts of PC (32:1) and PC (30:0) of the recurrence group were significantly higher than those of the non-recurrence group (Fig 1C). Principal component analysis also shows that PC (32:1) and PC (30:0) were selected as the peak combination to distinguish the recurrence group from the non-recurrence group (S3 Fig). PC (32:1) separated the recurrence and non-recurrence groups clearly whereas PC (30:0) did not. (Fig 1A and 1D). Statistical power of significance in the difference of signal intensity at *m/z* 732.5 was calculated as 99.9% based on power analysis for 3 recurrent cases and 6 non-recurrent cases. Moreover, the threshold value of the ratio of PC (32:1) to PC (38:6) for predicting recurrence could be set between 3.1 and 5.8. Therefore we propose PC (32:1) as the most powerful candidate for the predictive factor of TNBC recurrence. PC (32:1) and PC (30:0) have been reported to be associated with poor prognosis in breast cancer without classification according to intrinsic subtype [14]. As TNBC contains higher amounts of PC (32:1) and PC (30:0) compared with estrogen receptor positive and HER2 type breast cancers [13], this may indicate a poor prognosis for all TNBC types compared to other subtypes. The difference in the FA composition of PC between several breast cancer cells and the association with a metastatic potential have also been previously reported [15]. However, phospholipids and lipid metabolism of cultured cancer cells were reported to be different from those of solid tumors [16]. In the present study, we used human breast cancer tissues obtained from primary lesions only of patients with TNBC. Hence, we propose a powerful candidate for the predictive factor of recurrence in patients with TNBC. The prediction of recurrence in patients with TNBC would make it possible to provide beneficial personalized therapy. A future prospective study investigating whether the personalized therapy based on PC (32:1) intensity improves the prognosis of patients with TNBC would be necessary for factor validation. Moreover, the prediction of recurrence in patients with TNBC would make it possible to rapidly initiate treatment for recurrence, followed by an intensive postsurgical follow-up. A prospective study investigating whether intensive follow-up improves the prognosis of patients with TNBC with a high intensity of PC (32:1) would also be required.

PC (32:1) and PC (30:0) were more abundant in the cancer epithelial area compared to the stroma surrounding cancer in the recurrence group (Fig 2). Therefore, PC (32:1) and PC (30:0) were considered as abnormal lipid metabolism-generated metabolites that promote cancer metastasis. Compared to the PC content in non-malignant tissue, PC (32:1) and PC (30:0) were reported to accumulate in breast cancer tissues [9]. The abundance of PC (32:1) in the recurrence group is consistent with the results of a previous study on the malignant transformation of breast cancer. Furthermore, the accumulation of PC (32:1) and PC (30:0) was reported in invasive breast cancer, compared with non-invasive carcinoma [9]. This previous report also indicated that PC (32:1) and PC (30:0) were associated with the malignant transformation of breast cancer and is in agreement with our results. Ki67 is considered as a cellular marker for proliferation and a prognostic factor for breast cancer [4]. Correlation analysis revealed negative correlation between the mean value of averaged signal intensity of PC (38:6) at *m/z* 806.5 and Ki67 (S4 Fig).

PC (32:1), PC (30:0), and PC (38:6) can be assigned as PC (16:0/16:1), PC (16:0/14:0), and PC (16:0/22:6) according to a previous study, respectively [17]. The ratio of PC (16:0/16:1) to PC (16:0/22:6) was significantly higher in the recurrence group than it was in the non-recurrence group (Fig 1D), which can be explained by an increased level of FA (16:1) in the cancer epithelial area of the recurrence group. One possible reason why PC (16:0/16:1) containing FA (16:1) is increased in the recurrence group might be the up-regulation of stearoyl-CoA desaturase-1 (SCD1). SCD1, which converts saturated FA (SFA) to mono-unsaturated FA (MUFA), has been reported to be up-regulated in breast cancer with abundant PC (16:0/16:1) [18]. High SCD1 expression is associated with poor prognosis in patients with breast cancer [19]. As the inhibition of SCD 1 has been reported to reduce the proliferation and survival of cancer cells and, therefore, SCD 1may represent a suitable therapeutic target [20]. In the present study, there were no differences between the two groups regarding PC (34:1), which was considered to contain FA (16:1) (S1 and S2 Figs). *In vivo* phospholipids contain substantially more FA (16:0), FA (18:0), and FA (18:1) than FA (16:1) [21]. PC (34:1) might exist as PC (16:0/18:1) rather than as PC (16:1/18:0). Therefore, PC (34:1) might not increase in the recurrence group even if there were abundant FA (16:1). Immunohistochemical staining of SCD1 was performed using a modified protocol as previously reported [18]; the detailed method of SCD1 staining is shown in S1 Methods. Correlation between high PC (32:1) and stearoyl-CoA desaturase expression was not found (S5 Fig). Although there was no difference between the amount of SCD1 protein of the recurrence group and that of the non-recurrence group, SCD1 might have been activated more strongly in the recurrence group. As a lipidomic approach was applied in this study, we were able to identify a potential factor to predict recurrence in TNBC. The other possible explanation for the increased PC (16:0/16:1) in the recurrence group might be the up-regulation of lyso-phosphatidylcholine acyltransferase 1 (LPCAT1). PC can be synthesized by *de novo* and remodelling pathways [22]. In the remodelling pathway, LPCAT 1 is a key enzyme for the production of PC from lyso-phosphatidylcholine (LPC) by re-acylation, and its expression has been reported to increase in breast cancer with shorter survival rates [23]. Moreover, LPCAT1 is reported to be selective for LPC (16:1) and up-regulated LPCAT 1 increased PC (16:0/16:1) [24]. High expression levels of SCD1 and LPCAT1 might increase PC (16:0/16:1) levels and might be associated with poor prognosis. In the future, PC (32:1) may serve as a predictive factor of the therapeutic effect of inhibitors of SCD 1 and LPCAT 1 for treatment of patients with TNBC; further studies on PC profiles and related enzymes will be required to verify this possibility.

The current study contains two limitations, a low number of sample and a short follow-up period. TNBC includes heterogeneous groups of cancer subtypes. Taking the parent population into account, increasing the sample number is an important requirement to determine whether PC (32:1) is common to the recurrence of all case of TNBC. The median follow-up period of the patients was two years in the present study; therefore, relapses later than two years might not have been included in the present analysis. It is thus necessary to increase the number of samples and follow-up over a longer term in order to validate PC (32:1) as a predictive factor of recurrence in patients with TNBC.

In this study, we identified a candidate predictive factor of TNBC recurrence. Specifically, we showed that recurrent TNBC contains s significantly higher amount of PC (32:1) that is associated with the remodeling pathway in PC metabolism.

## Methods

### Ethics statement

The experiments in this study were approved by the Ethics Committee of the Hamamatsu University School of Medicine. Written informed consent was obtained from all patients before surgery. All experiments were performed in accordance with the approved guidelines. The patients consented to cooperate after they were informed that they would not incur any disadvantage, that they could resign from the study, that the researchers were obliged to protect their privileged information, and that their identities would not be revealed.

### Human tissue samples and clinicopathological data

All patients undergoing breast cancer surgery at the Hamamatsu University Hospital were recruited between January, 2011 and July, 2013. Tissue samples of primary lesions were collected at the time of surgery at Hamamatsu University Hospital. The primary lesions were histologically diagnosed as breast cancer by experienced pathologists. The size of the primary lesion ranged from 9–120 mm in the greatest dimension (S2 Table). The surface area of the sample sections was measured using the NanoZoomer Digital Pathology Virtual Slide Viewer (Hamamatsu photonics, Shizuoka, Japan) and ranged from 4.6–37.2 mm^2^ (S2 Table). Of 125 patients, 9 were all negative for immunohistochemical staining of estrogen receptor, progesterone receptor, and HER2. The collected samples were rapidly frozen in cooled *n*-hexane at −80°C. Patients’ clinical records were reviewed to ensure there were no distant metastases before surgery and check the recurrence. We defined recurrence as a new imaging-based finding of a distant metastasis.

### Chemicals

2, 5-dihydroxyacetophenone (DHAP) was purchased from Bruker Daltonics (Billerica, MA, USA). Ammonium sulfate was obtained from Kanto Chemicals (Tokyo, Japan). Liquid chromatography (LC)/MS-grade ultrapure water and high performance liquid chromatography-grade ethanol were obtained from Wako Pure Chemical Industries (Osaka, Japan).

### Sample preparation

The samples were prepared with reference to the methods used in a previous study [18]. Frozen tissues were cut to10- μm -thick using a cryostat (CM1950, Leica Biosystems, Wetzlar, Germany), and thaw-mounted on an electro-conductive glass slide coated with indium-tin-oxide (Bruker Daltonics) for the MALDI-IMS and MS/MS analyses at −20°C. The samples were stored at –80°C until analysis. Serial sections of the samples were mounted onto a Matsunami adhesive silane (MAS) coated glass (Matsunami Glass Industry, Osaka, Japan) and stained with H&E. The serial sections were fixed with 4% paraformaldehyde solution for 30 min, washed with distilled water, treated with Mayer’s hematoxylin for 5 min, and then washed twice with distilled water. The sections were treated with eosin for 1 min, washed twice with distilled water, and then the fixed and stained sections were dehydrated by immersion in 100% ethyl alcohol and xylene in order five times each for 10 s each time. The DHAP solution (5 mg/ml in 50% ethanol/125 mM ammonium sulfate, v/v) was used as the matrix solution as previously reported[25]. Then, 4ml of the matrix solution was uniformly sprayed on the surface of the tissue section using a 0.2-mm nozzle caliber airbrush (Procon Boy FWA Platinum; Mr. Hobby, Tokyo, Japan). The distance between the nozzle tip and the tissue surface was maintained at 20 cm.

### MALDI-IMS and MALDI-MS/MS analyses

The MALDI-IMS was performed using a high-resolution microscopic imaging mass spectrometer (Mass Microscope, a prototype of the iMScope, Shimadzu, Kyoto, Japan) with an atmospheric pressure-MALDI and quadrupole ion trap time-of flight analyser, equipped with a 355-nm Nd:YAG laser at 1000 Hz repetition rate, and controlled using the Imaging MS Solution™ program (Shimadzu, Kyoto, Japan) [17]. The mass spectra were acquired at *m/z* 700–900 in the positive ion mode with the scan pitch of 50 μm and a laser diameter of 5 μm. Images for each peak were obtained using the Imaging MS Solution™ based on signal intensity displayed in the mass spectra. All MS/MS experiments on the breast cancer tissue were performed using the recurrent case No. 7 samples using the same instrument used for the MALDI-IMS with reference to a previous study [26]. The isolation window was set to ± *m/z* 1.0 for each precursor ion, and the scan pitch was set to 5 μm, and the repeating number was 1. The other setting status was the same as that used in the MALDI-IMS analysis.

### Data analysis

The spectra and averaged signal intensities of the ROIs were analyzed using the Imaging MS Solution™ program. Averaged signal intensity is obtained by averaging signal intensity inside a single ROI. All the spectra were normalized to their own total ion currents according to a previous study to eliminate uncontrolled experimental factors [17]. The ROIs were set to the areas of approximately 1000 × 1000 μm in the cancer epithelial area yielding total 400 data points, as identified by referring to the H&E stained sections. The histological diagnosis was performed by examining the H&E-stained serial tissue sections. Peaks were selected with a signal-to-noise ratio threshold of 5, and the mean value of the averaged signal intensity of three ROIs yielding total 1200 points in total was compared between the recurrence and the non-recurrence groups. Sample No.6 was too small to be set to three areas of 1000 × 1000 μm in the cancer epithelial area and, therefore the mean value of the averaged signal intensity of two ROIs yielding total 800 points in total was compared. The statistical analysis was performed using the Mann-Whitney U test using the statistical package for the social science (SPSS) program-version21.0 (IBM, Armonk, NY, USA). P < 0.05 was considered statistically significant. The experiment was designed as follows; assuming significant difference with P < 0.05 and 99% for statistical power, we aimed to find signal with effect size larger than 4.0. Power analysis was carried out using the statistical analysis tool R version 3.2.4: A Language and Environment for Statistical computing (R Foundation for Statistical Computing, Vienna, Austria) and package ‘pwr’ version1.2–1. Unsupervised multivariate analysis, principal component analysis was also performed using ClinProTools 2.2. (Bruker Daltonics) [17]. Variance in high-dimensional molecular composition was contracted as three-dimensional scatter plots of principal components. Clustering of sample groups in the scatter plot shows that the sample groups are distinguishable by the combination of principal components. A couple of signals in a mass spectrum were automatically chosen taking the loading score into account by the software. The molecular identification of the observed peaks of interest was performed using the Human Metabolome Database (http://www.hmdb.ca/spectra/ms/search) and MS/MS analysis.

## Supporting information

S1 Table Observed peaks and lipid species. (DOCX)

S2 Table Primary lesion and sample sizes. (DOCX)

S1 Fig Comparison of averaged signal intensities of 15 peaks between non-recurrence and recurrence groups. Scatter plots of averaged signal intensities at *m/z* 703.5, 756.5, 758.5, 760.5, 762.5, 780.5, 782.5, 784.5, 786.5, 788.5, 808.5, 810.5, 812.5, 814.5, and 816.5 of recurrence and non-recurrence groups are presented. No significant differences occurred in any peaks between both groups in any peaks using the Mann-Whitney U test. NS, not statistically significant.
(TIF)

S2 Fig Ion distributions of 15 peaks for all 9 samples. MALDI-IMS analysis shows the ion distributions at *m/z* 703.5, 756.5, 758.5, 760.5, 762.5, 780.5, 782.5, 784.5, 786.5, 788.5, 808.5, 810.5, 812.5, 814.5, and 816.5. Both groups show high signal intensities at *m/z* 760.5 in the cancer epithelial area. Scale bar = 1000 μm. MALDI-IMS: matrix-assisted laser desorption/ionization–imaging mass spectrometry, H&E: hematoxylin and eosin.
(TIF)

S3 Fig PCA analysis of the non-recurrence and recurrence groups. (A) Scatter plots of principal component. (B) 3-D scatter plots of principal component. (C) Scatter plots of *m/z* 706.5 vs 732.5. PCA revealed that the non-recurrence and recurrence groups were most distinguishable with the combination of *m/z* 706.5 vs 732.5. Axes in the scatter plots are shown as arbitrary units. Green: non-recurrence group, red: recurrence group. PCA: principal component analysis.
(TIF)

S4 Fig Correlation analysis between PC (30:0), PC (32:1), PC (32:0), and PC (38:6). Correlation analysis revealed negative correlation between the mean value of averaged signal intensity of PC (38:6) at *m/z* 806.5 and Ki67.
(TIF)

S5 Fig Comparison of the signal intensity of PC (32:1) and the intensity of SCD1 staining. (A) Immunohistochemical staining of stearoyl-CoA desaturase-1. Scale Bar: 500 μm. (B) Comparison of the signal intensity of PC (32:1) and the intensity of SCD1 staining. Correlation between the signal intensity of PC (32:1) and the intensity of SCD1 staining was not found.
(TIF)

S1 Data Data on each averaged signal intensity of ROIs for each *m*/*z*. (XLSX)

S1 Methods Immunohistochemical staining of SCD1. (DOCX)
