# Supplementary material for: Recurrent triple-negative breast cancer (TNBC) tissues contain a higher amount of phosphatidylcholine (32:1) than non-recurrent TNBC tissues
Source: PLoS One. 2017 Aug 23;12(8):e0183724. doi: 10.1371/journal.pone.0183724 (PMC5568295; doi:10.1371/journal.pone.0183724)
Supplement: S1 Table — (DOCX) [file pone.0183724.s001.docx]

**Table S1. Observed peaks and lipid species**

SM: sphingomyelin PC: phosphatidylcholine

HMDB: Human Metabolome Database (http://www.hmdb.ca/spectra/ms/search)

| *m/z* | Lipid | Reference |
| --- | --- | --- |
| 703.5 | [SM(d18:1/16:0)+H]^+^ | 1,3 |
| 706.5 | [PC (30:0)+H]^+^ | 2 |
| 732.5 | [PC (32:1)+H]^+^ | 2,3 |
| 734.5 | [PC (32:0)+H]^+^ | 1,3 |
| 756.5 | [PC (34:3)+H]^+^ | 3 |
| 758.5 | [PC (34:2)+H]^+^ | 3 |
| 760.5 | [PC (34:1)+H]^+^ | 1,3 |
| 762.5 | [PC (34:0)+H]^+^ | 3 |
| 780.5 | [PC (36:5)+H]^+^ | HMDB |
| 782.5 | [PC (36:4)+H]^+^ | 3 |
| 784.5 | [PC (36:3)+H]^+^ | 3 |
| 786.5 | [PC (36:2)+H]^+^ | 1,3 |
| 788.6 | [PC (36:1)+H]^+^ | 1,3 |
| 806.5 | [PC (38:6)+H]^+^ | 3 |
| 808.5 | [PC (38:5)+H]^+^ | HMDB |
| 810.5 | [PC (38:4)+H]^+^ | 3 |
| 812.5 | [PC (38:3)+H]^+^ | HMDB |
| 814.6 | [PC (38:2)+H]^+^ | 3 |
| 816.6 | [PC (38:1)+H]^+^ | 3 |

Reference

1, Chughtai, K., Jiang, L., Greenwood, T. R., Glunde, K. & Heeren, R. M. Mass spectrometry images acylcarnitines, phosphatidylcholines, and sphingomyelin in MDA-MB-231 breast tumor models. J. Lipid Res. 54, 333-344, doi:10.1194/jlr.M027961 (2013).

2, Kang, H. S. et al. Protein and lipid MALDI profiles classify breast cancers according to the intrinsic subtype. BMC Cancer. 11, 465, doi:10.1186/1471-2407-11-465 (2011).

3, Delvolve, A. M., Colsch, B. & Woods, A. S. Highlighting anatomical sub-structures in rat brain tissue using lipid imaging. Anal. Methods. 3, 1729-1736, doi:10.1039/C1AY05107E (2011).
