# Supplementary material for: Recurrent triple-negative breast cancer (TNBC) tissues contain a higher amount of phosphatidylcholine (32:1) than non-recurrent TNBC tissues
Source: PLoS One. 2017 Aug 23;12(8):e0183724. doi: 10.1371/journal.pone.0183724 (PMC5568295; doi:10.1371/journal.pone.0183724)
Supplement: S2 Table — (DOCX) [file pone.0183724.s002.docx]

**S2 Table. Primary lesion and sample sizes.**

| No | Primary lesion (mm) | Sample (mm^2^) |
| --- | --- | --- |
| 1 | 27 | 21 |
| 2 | 51 | 11.2 |
| 3 | 17 | 26.6 |
| 4 | 18 | 14.8 |
| 5 | 9 | 4.6 |
| 6 | 25 | 10.8 |
| 7 | 27 | 8.4 |
| 8 | 30 | 37.2 |
| 9 | 160 | 14 |

The size of the primary lesion is presented as invasive size in the greatest dimension. The size of the sample is presented as the surface area of the sample sections.
