# Supplementary figures and images for: Recurrent triple-negative breast cancer (TNBC) tissues contain a higher amount of phosphatidylcholine (32:1) than non-recurrent TNBC tissues
Source: PLoS One. 2017 Aug 23;12(8):e0183724. doi: 10.1371/journal.pone.0183724 (PMC5568295; doi:10.1371/journal.pone.0183724)

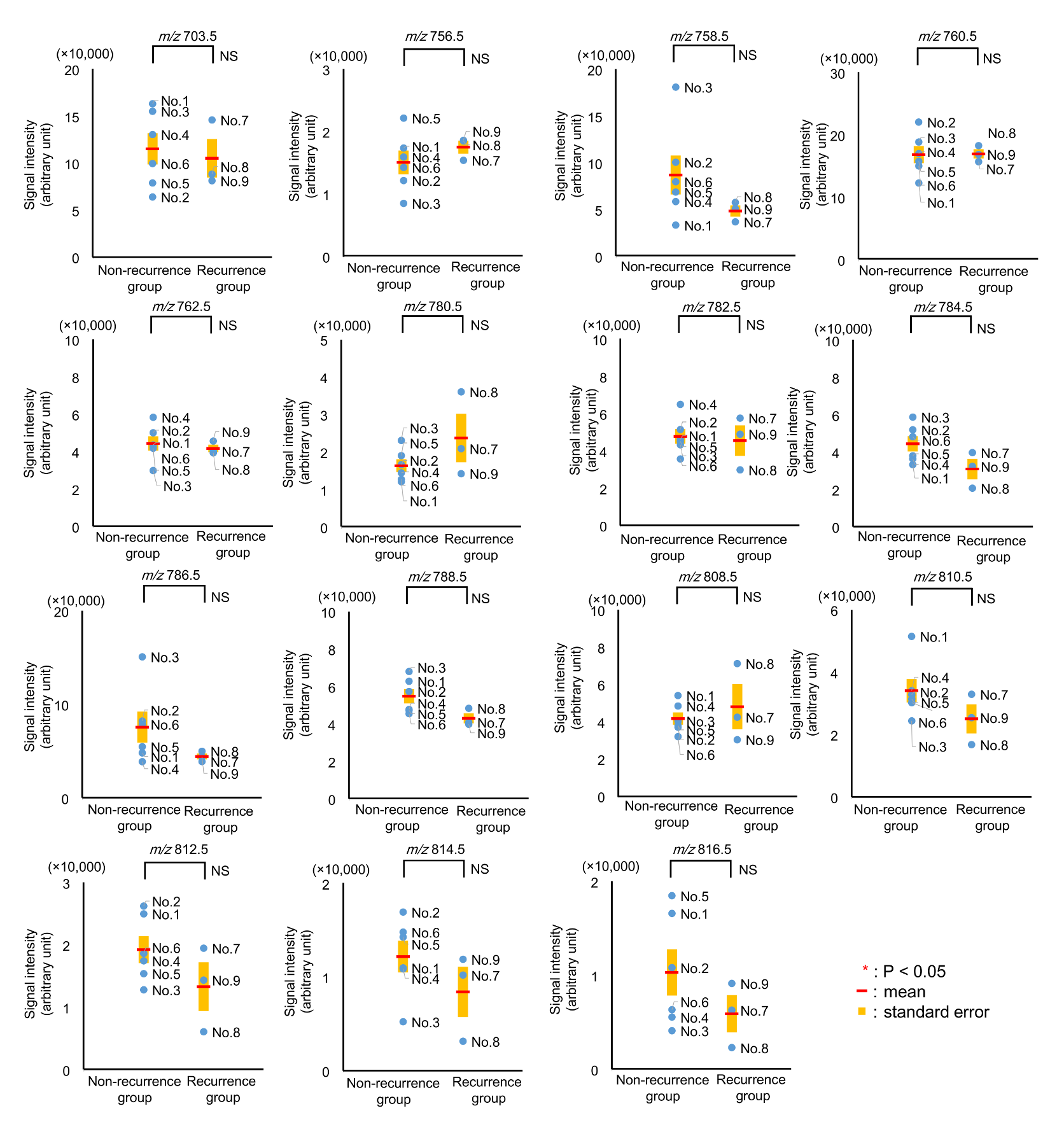

Supplement: S1 Fig — Scatter plots of averaged signal intensities at m/z 703.5, 756.5, 758.5, 760.5, 762.5, 780.5, 782.5, 784.5, 786.5, 788.5, 808.5, 810.5, 812.5, 814.5, and 816.5 of recurrence and non-recurrence groups are presented. No significant differences occurred in any peaks between both groups in any peaks using the Mann-Whitney U test. NS, not statistically significant. (TIF) [file pone.0183724.s003.tif]

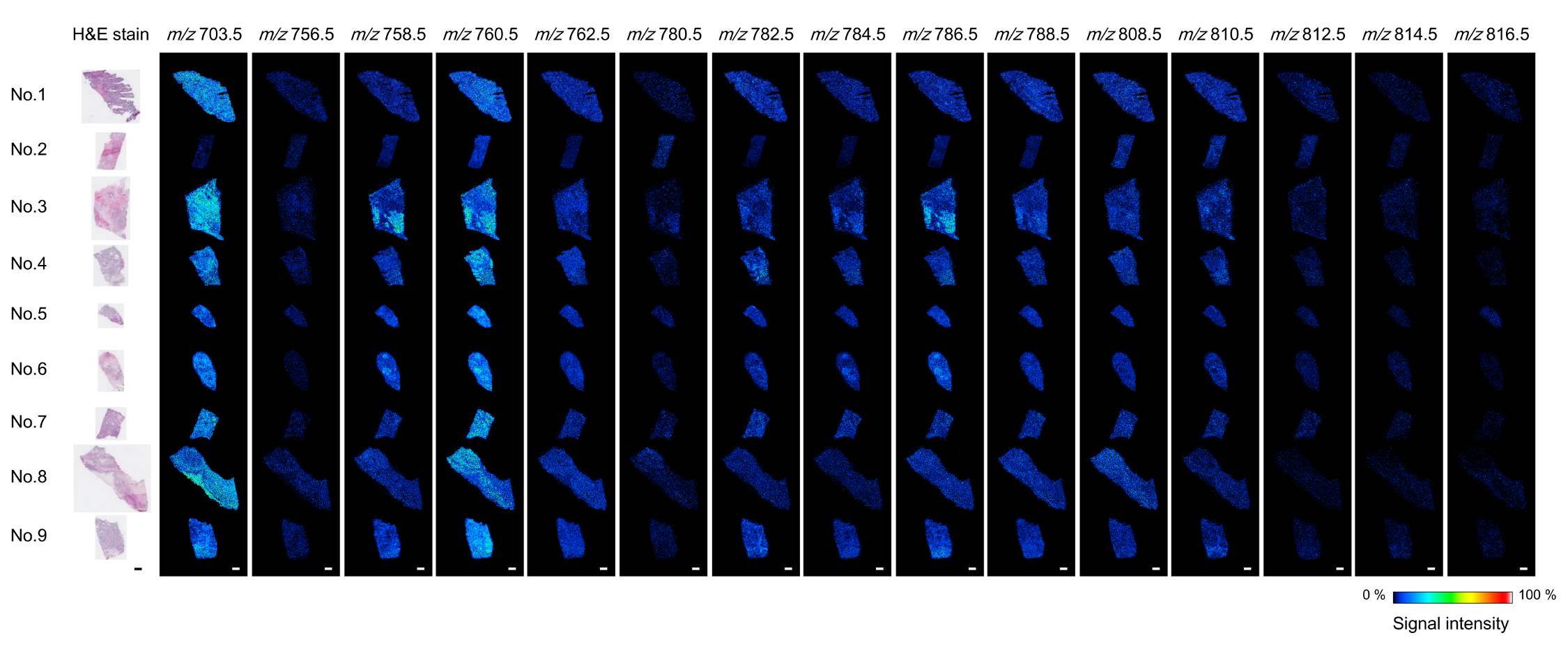

Supplement: S2 Fig — MALDI-IMS analysis shows the ion distributions at m/z 703.5, 756.5, 758.5, 760.5, 762.5, 780.5, 782.5, 784.5, 786.5, 788.5, 808.5, 810.5, 812.5, 814.5, and 816.5. Both groups show high signal intensities at m/z 760.5 in the cancer epithelial area. Scale bar = 1000 μm. MALDI-IMS: matrix-assisted laser desorption/ionization–imaging mass spectrometry, H&E: hematoxylin and eosin. (TIF) [file pone.0183724.s004.tif]

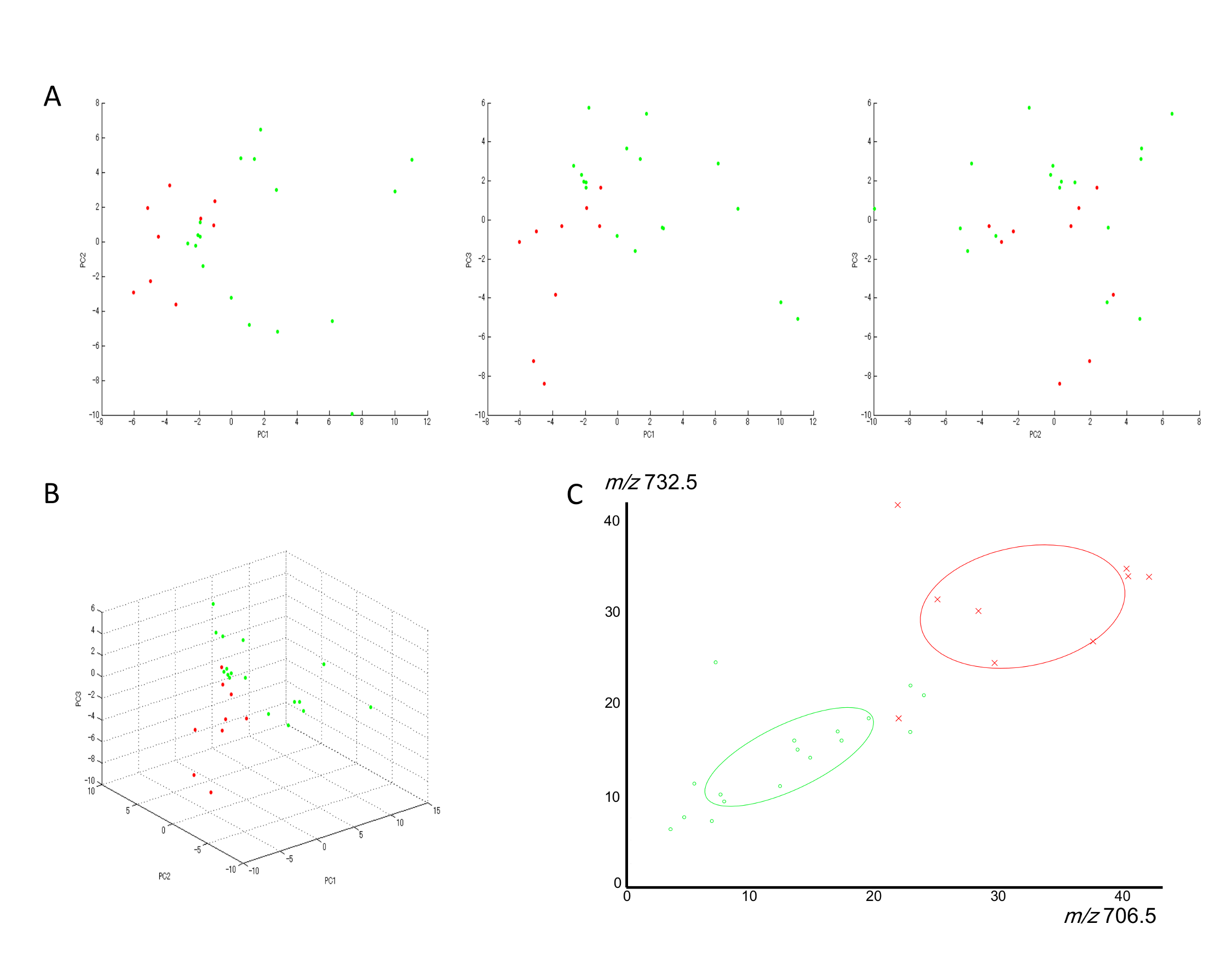

Supplement: S3 Fig — (A) Scatter plots of principal component. (B) 3-D scatter plots of principal component. (C) Scatter plots of m/z 706.5 vs 732.5. PCA revealed that the non-recurrence and recurrence groups were most distinguishable with the combination of m/z 706.5 vs 732.5. Axes in the scatter plots are shown as arbitrary units. Green: non-recurrence group, red: recurrence group. PCA: principal component analysis. (TIF) [file pone.0183724.s005.tif]

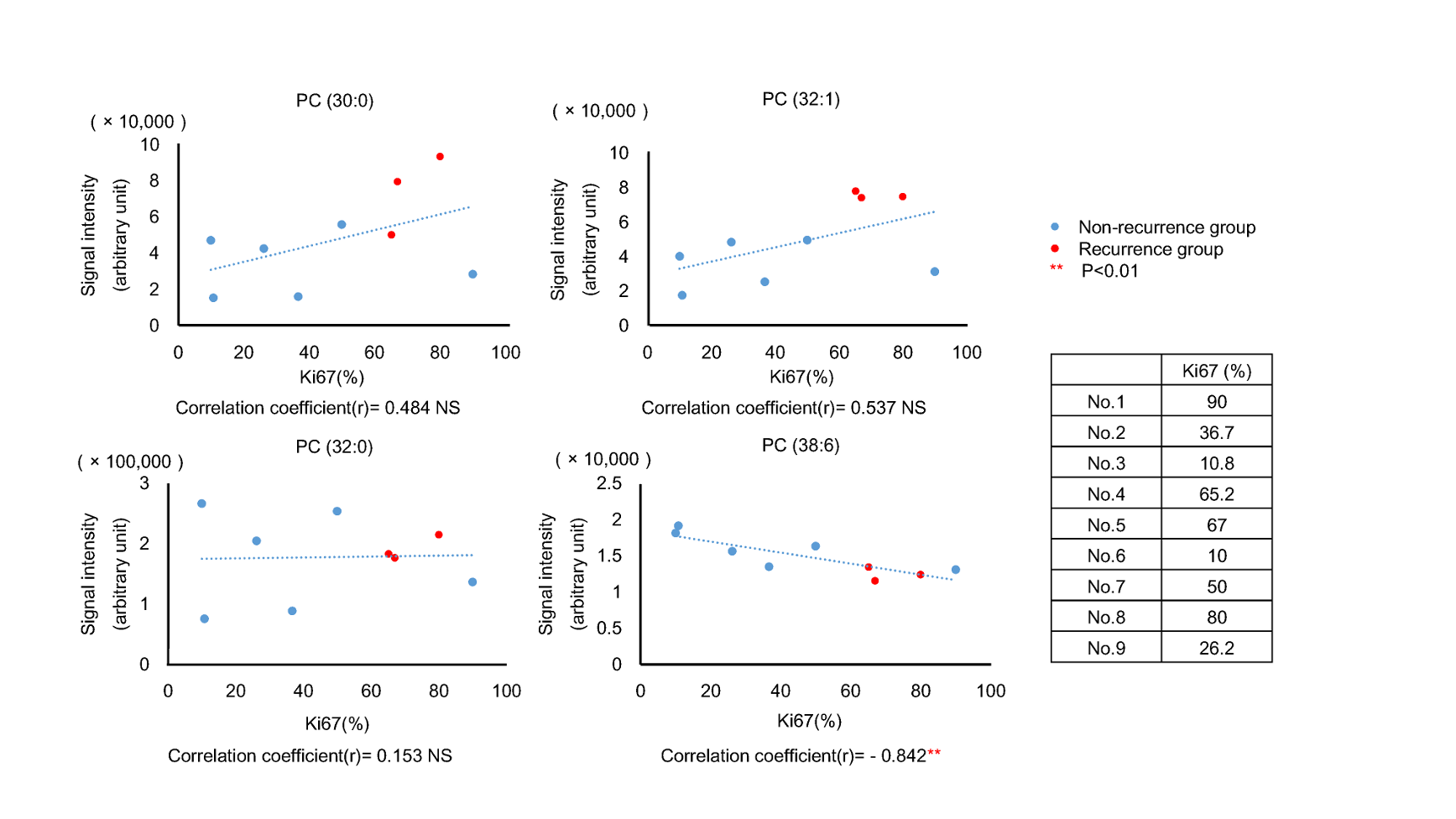

Supplement: S4 Fig — Correlation analysis revealed negative correlation between the mean value of averaged signal intensity of PC (38:6) at m/z 806.5 and Ki67. (TIF) [file pone.0183724.s006.tif]

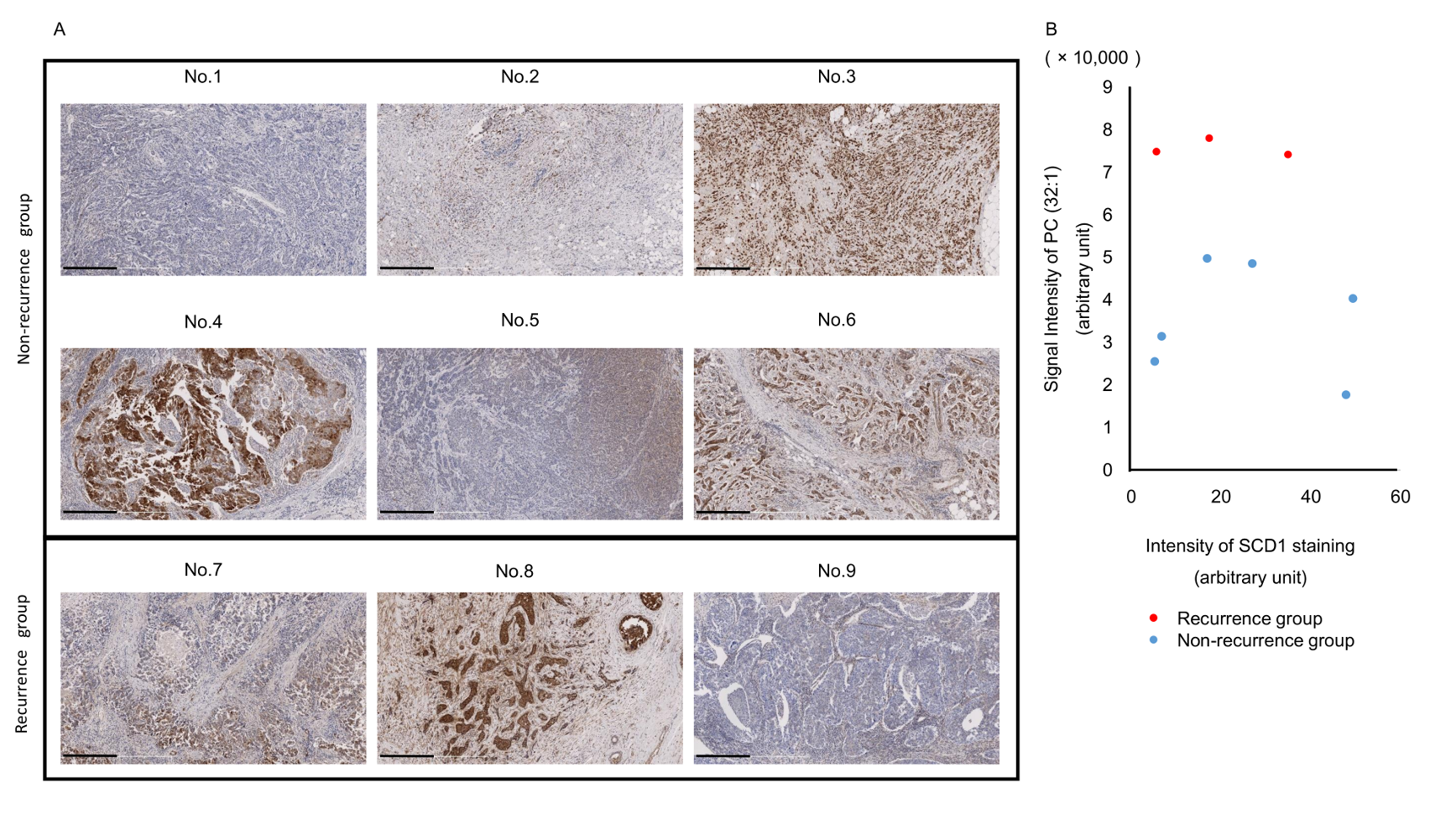

Supplement: S5 Fig — (A) Immunohistochemical staining of stearoyl-CoA desaturase-1. Scale Bar: 500 μm. (B) Comparison of the signal intensity of PC (32:1) and the intensity of SCD1 staining. Correlation between the signal intensity of PC (32:1) and the intensity of SCD1 staining was not found. (TIF) [file pone.0183724.s007.tif]
