# Supplementary material for: Recurrent triple-negative breast cancer (TNBC) tissues contain a higher amount of phosphatidylcholine (32:1) than non-recurrent TNBC tissues
Source: PLoS One. 2017 Aug 23;12(8):e0183724. doi: 10.1371/journal.pone.0183724 (PMC5568295; doi:10.1371/journal.pone.0183724)
Supplement: S1 Methods — (DOCX) [file pone.0183724.s009.docx]

**Immunohistochemical staining**

Part of tissue blocks, fixed with formalin and embedded with paraffin, were cut with 3 μm-thick and mounted on a slide glass. The sections were deparaffinized in xylene and rehydrated thorough a descending series of ethanol. The sections were heated in Tris- ethylenediamintetraacetic acid buffer (pH 9) for 40 min. at 95°C for antigen retrieval and cooled for 30 min. at room temperature. After washing them several times in phosphate buffered saline, endogenous peroxidase was blocked in 3% H_2_O_2_ in methanol for 5 min. They were then incubated with mouse monoclonal antibody against SCD1 (1:50; GeneTex, Irvine, CA, USA) for 30 min. The section were visualized by using a peroxidase/DAB (Dako ChemMate EnVision kit K5007; Dako, Glostrup, Denmark) with incubation of Envision/HRP, rabbit/mouse for 30 min. and DAB+chromogen for 5min. Immunohistochemical procedure was performed using automated staining equipment (Histostainer; Nichirei Biosciences Inc., Tokyo, Japan), according to the manufacturer’s instructions. Nuclei were counterstained with Mayer’s hematoxylin. Basal epithelial cells in the mammary gland were used as an inner negative control. Sebaceous grands in skin of breast were used as an inner positive control. The expression levels for the positive cells were assessed using Image J (NIH, Bethesda, MD, USA).
